# Mathematical models of nitrogen-fixing cell patterns in filamentous cyanobacteria

**DOI:** 10.3389/fcell.2022.959468

**Published:** 2022-09-16

**Authors:** Pau Casanova-Ferrer, Javier Muñoz-García, Saúl Ares

**Affiliations:** ^1^ Grupo Interdisciplinar de Sistemas Complejos (GISC), Madrid, Spain; ^2^ Departamento de Matemáticas, Universidad Carlos III de Madrid, Leganés, Spain; ^3^ Centro Nacional de Biotecnologia (CNB), CSIC, Madrid, Spain

**Keywords:** pattern formation, cyanobacteria, heterocyst differentiation, nitrogen fixation, gene regulatory networks, activator-inhibitor, reaction-diffusion

## Abstract

The *Anabaena* genus is a model organism of filamentous cyanobacteria whose vegetative cells can differentiate under nitrogen-limited conditions into a type of cell called a heterocyst. These heterocysts lose the possibility to divide and are necessary for the filament because they can fix and share environmental nitrogen. In order to distribute the nitrogen efficiently, heterocysts are arranged to form a quasi-regular pattern whose features are maintained as the filament grows. Recent efforts have allowed advances in the understanding of the interactions and genetic mechanisms underlying this dynamic pattern. Here, we present a systematic review of the existing theoretical models of nitrogen-fixing cell differentiation in filamentous cyanobacteria. These filaments constitute one of the simplest forms of multicellular organization, and this allows for several modeling scales of this emergent pattern. The system has been approached at three different levels. From bigger to smaller scale, the system has been considered as follows: at the population level, by defining a mean-field simplified system to study the ratio of heterocysts and vegetative cells; at the filament level, with a continuous simplification as a reaction-diffusion system; and at the cellular level, by studying the genetic regulation that produces the patterning for each cell. In this review, we compare these different approaches noting both the virtues and shortcomings of each one of them.

## 1 Introduction

Pattern formation is extremely relevant in embryonic development because it allows for precise periodic spatial differentiation of certain cells or groups of cells. An important question is how a pattern, and, therefore, heterogeneity, is produced from a homogeneous state, given that embryos develop from a single cell. Another intriguing feature is that patterning must be robust enough to ensure reliability, given that embryo development is a highly reproducible process. Additionally, the widespread action of pattern formation in all organisms and different levels of development seems to point to the existence of simple intrinsic mechanisms capable of acting with widely different elements.

The reaction-diffusion system, presented by Turing in his seminal work (Turing, 1952), constitutes a simple model capable of forming spatial patterns starting from a homogeneous state. Turing considers a ring of equivalent cells that generate a couple of diffusible morphogens whose production depends on the concentrations of both of them. He realized, through a linear perturbation analysis, that, while the system starts homogeneous, slight perturbations in the diffusion of morphogens are reinforced and create “waves” of morphogens in the cell ring. This reinforcement is caused because when a cell sends more inhibitor to its neighboring cells than what it receives, the neighboring cells produce less inhibitor. This further reduces the flux of inhibitor that enters the cell, which, in turn, increases inhibitor production and its flux to the neighboring cells. This feedback loop produces waves of morphogens that can drive the system to a heterogeneous state if system parameters are capable of sustaining the perturbation out of the linear regime. Furthermore, if there are more than two diffusible morphogens, the heterogeneous state can be oscillatory. The general condition that allows these instabilities to form is the combination of an activator and a more diffusible inhibitor. The particular ratio between the diffusion rates is highly dependent on the reaction system that regulates these morphogens (Gierer and Meinhardt, 1972). This fine-tuning required for the pattern fixation questions the biological feasibility of this mechanism because it makes the system susceptible to small changes in parameter values that would greatly alter its behavior.

These types of biological pattern–forming systems were further extensively studied by Meinhardt (2008) and fully theoretically fledged out by Murray (2003). A state-of-the-art discussion on Turing’s ideas, their development, and some system examples can be found in a research study on this same Special Topic issue (Lacalli, 2022). Subsequently, Murray’s analysis has been expanded by considering reaction-diffusion systems in continuous growing domains, observing that depending on the characteristics of the growth, it can produce more robust pattern formation or add difficulties to it (Crampin et al., 1999; Barrass et al., 2006). Finally, the limiting case in which the activator does not diffuse cannot create a stable stationary pattern; therefore, the emergent patterns are always of a dynamical nature (Marciniak-Czochra et al., 2017). The incorporation of mechano-chemical feedback can mediate the reinforcement and consequent fixation of the pattern through a morphological change that affects the diffusion of the inhibitory morphogen (Brinkmann et al., 2018).

When talking about these reaction-diffusion systems, it is important to remember that the insights from linear stability analysis, usually invoked to determine whether a system can form a stable pattern or not, can be deceiving: the dispersion relation close to a homogeneous fixed point can sometimes be very helpful, but also deceiving once full nonlinearities kick in. For this reason, classical rules for pattern formation based on linear analysis are better understood as applying to pattern inception, given that the study of linear perturbations and the stabilization of a final pattern is a process where nonlinearities cannot, in general, be neglected (Smith and Dalchau, 2018). For instance, against classical thinking, systems with equally diffusing signals can make stable patterns (Marcon et al., 2016). In this framework, it is clear that events such as domain growth (Raspopovic et al., 2014), discrete nature of the system (Nakamasu et al., 2009), or separation of timescales for the action of different molecular species can all play a role to shape the formation and maintenance of patterns.

**FIGURE 1:**
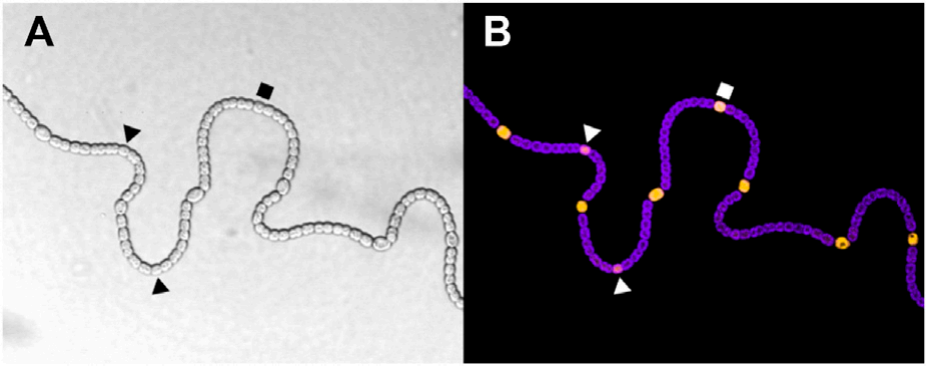
**(A)** Wild type *Anabaena* PCC 7120 filament grown in conditions of nitrogen deprivation showing vegetative cells and heterocysts. **(B)** Florescence images characterize the differentiation stage of each cell. The purple florescence is associated with the thylakoid membranes (and therefore with the photosynthesis), while the yellow florescence marks NsiR1 expression, which is described as an early marker of differentiation in Muro-Pastor (2014). Intermediate stages of developing heterocysts are indicated by polygonal shapes. Images are courtesy of Alicia Muro-Pastor.

All these characteristics are relevant for the study of pattern formation in the filamentous cyanobacterium *Anabaena* (Figure 1). The cells of the filament exchange nutrients and react as a whole to environmental changes. One could argue that, while each cell is still a unicellular organism, the filament is located close to the transition to multicellular organization. This is especially evident when the filament is placed in conditions of nitrogen deprivation. Under these conditions, the filament undergoes a dynamical differentiation process that differentiates roughly one in every ten cells into nitrogen-fixing heterocysts in a quasi-regular pattern that is maintained as the filament keeps growing (Flores and Herrero, 2010). This patterned differentiation constitutes an example of specialization, cooperation, and distribution of labor because, while the vegetative cells keep producing carbon through photosynthesis, the heterocysts fix environmental nitrogen into organic forms that can be assimilated by all cells. Thus, for the filament to subsist, both end products must be shared and diffused through the filament to the cells that are not capable of synthesizing them. While previous reviews have already compiled the current theories about heterocyst pattern formation (Flores and Herrero, 2010; Herrero et al., 2016; Harish and Seth, 2020; Zeng and Zhang, 2022), in this review, we systematically discuss the different mathematical and computational frameworks that have been used to model the physics of cell differentiation and pattern formation in this system.

## 2 Diffusion models of an inhibitor exported from heterocysts

First, we present research works that attempted to model the patterned distribution of heterocysts without any explicit genetic regulation. With this aim, these models only considered gradients in nitrogen concentration or some inhibitory signal originating from heterocysts.

An early attempt of modeling the patterning of heterocyst differentiation in cyanobacteria filaments came at a time when, while the biological role of heterocysts was not well-defined (Fay et al., 1968), it was already stated that heterocysts seemed to inhibit the formation of new ones (Wolk, 1967). The model presented by Baker and Herman (1972) consisted of an integer linear cell array simulator which allowed cell-to-cell diffusion of an inhibitory product and division of vegetative cells. Due to computational limitations at the time, concentrations were modeled as integer numbers, setting a discrete minimum change in concentration as a result. This model was used to test the hypothesis that cell division and differentiation are two competing processes in which, at the end of each cell cycle (quantified by a countdown), cells have to choose a fate depending on the inhibitor concentration. This simple model could obtain feasible distributions for heterocyst placement, but the code was heavily limited by having to work with integer concentrations. The model predicted a low threshold of the inhibitor to avoid differentiation, causing integer rounding to be comparable with concentration values. This low threshold was probably caused by the unrealistic assumption of an equal rate for cell–cell and cell–media diffusion, which impeded the formation of a well-defined inhibitory gradient in the filament.

Just a year after this first study, the group responsible for one of the initial experimental studies (Wolk, 1967) also presented theoretical results using a simulation code (Wolk, 1975). The authors considered that heterocyst placement was defined by a diffusible inhibitor whose concentration dynamics was expressed as:

$$\frac{\partial C\left(x,t\right)}{\partial t}=D\frac{\partial^{2}C\left(x,t\right)}{\partial x^{2}}-k\cdot C\left(x,t\right).$$ (1)

Here, *C* (*x*, *t*) is the concentration of the diffusible inhibitor at the point *x* in the time *t*, *D* is the diffusion constant, and *k* is the decay rate.

From this equation, the authors obtained an inhibitor diffusion root mean square distance for the closed (*k* = 0) and general systems by considering a discrete approximation with cells as distance units and the inhibitor generated from a point source. These two distances were used as alternative ways to define the range of inhibition that a heterocyst has over neighboring cells in the simulation. This simulation was a sequential random pick of non-inhibited vegetative cells that continued until all cells were inhibited or heterocysts. The solution for the general system agreed with the experimental distribution of distances between heterocysts better than the closed system. However, the closed system produced a slightly more uniform distribution, while presenting much longer intervals than the experimental data. This led the authors to propose two diffusion-based spacing mechanisms in which a heterocyst would appear on a cell sufficiently distant from preexisting heterocysts so that it has a concentration of the activator higher than some critical level. At the heart of this work was the initial idea that heterocyst differentiation is a purely stochastically driven process. Thus, control is only exerted through desensitization that protects the cells that are close to existent heterocysts against differentiation.

The same diffusive Eq. 1 was studied by De Koster and Lindenmayer (1987), obtaining two different analytical solutions (one continuous and another discrete). These solutions were compared with an improved version of the integer linear cell array simulator discussed previously. This version avoids some problems faced in the study by Baker and Herman (1972) by storing the concentration as a floating-point variable and eliminating the environment with the initialization of the filament already in equilibrium with two heterocysts in the extremes. Through this comparison, two biologically reasonable estimations were made: *D* = 0.14–0.39 μm^2^/s for the inhibitor diffusion constant, and *k* = 2.7–7.5 ⋅ 10^–4^ s^−1^ for the degradation rate, and an inaccurate estimation for the cell cycle, 7.25 h, which is known to be around 24 h.

Much later, Allard et al. (2007) proposed a series of models. While the first three models are discussed here, the last one is considered in the following section as it includes some genetic interactions. The initial work (Allard et al., 2007) compares the distribution of heterocysts obtained through random placement with one obtained with a model of nitrogen propagating over a filament with a continuous periplasm. In this model, vegetative cells consume nitrogen to grow, while heterocysts produce nitrogen that diffuses through the filament. When the nitrogen level of a vegetative cell reaches 0, the cell irreversibly commits to differentiation. Additionally, the cells grow at a constant rate and divide at a certain fixed division time for each cell. The model is initialized with a couple of heterocysts at the ends of the filaments and a randomly distributed growth rate for each cell. For this model to be able to reproduce the experimental distributions, the authors have to consider an immediate release of nitrogen after commitment in order to avoid the formation of multiple heterocysts. This work presents an opposing paradigm to the earlier ideas by Wolk (1967): while in the oldest work, there was a deterministic system of inhibition with a stochastic initiation of differentiation, this work includes a deterministic drive that starts the differentiation to explain *de novo* heterocyst formation. Nevertheless, the need to include a sizable immediate release of nitrogen once a cell is committed to differentiation to avoid the formation of clusters of heterocysts shows that some level of stochasticity is necessary. This stochasticity is represented here by the random distribution of growth rates along the filament. The heterogeneity of growth rates will decide which one of the cells, located in a nitrogen-deprived area, will consume faster its reserves and therefore become a heterocyst. This interplay between deterministic dynamics on a random heterogeneous system seems necessary to recover the observed experimental heterocyst spacing distributions and will be a common trait of most of the models presented below.

This model was expanded by Brown and Rutenberg (2012a) and Brown and Rutenberg (2012b) with the addition of a coupling between the growth and the available nitrogen in the cell and the possibility of nitrogen leakage into the media. Additionally, the commitment condition is also modified, and cells have to remain in complete nitrogen deprivation for a set time before they differentiate into heterocysts. This model is capable of reproducing the experimental placement of heterocysts (with a commitment time of 8 h) considerably better than a random placement and a partially random one where positions adjacent to heterocysts cannot differentiate. Nevertheless, the assumption that a heterocyst is capable of releasing a sizable amount of fixed nitrogen right after commitment is not biologically feasible, and would be substituted by genetic regulation in later research work (Brown and Rutenberg, 2014) (described in Section 3). The authors also obtained a relationship between filament growth rate and heterocyst frequency and found that growth rate presents a maximum for a certain value of heterocyst frequency (Brown and Rutenberg, 2012a). This maximal growth is similar for different placement strategies if nitrogen leakage is not considered in the model. However, if leakage over 1% is considered, the differences in the growth rate between strategies are relevant; the strategy of differentiation by nitrogen-starved cells, that produced the most realistic heterocyst distributions, is also the one that produces maximal growth (Brown and Rutenberg, 2012a).

Alternatively, Ishihara et al. (2015) considered a paracrine inhibitory signal that originated from the heterocysts instead of considering the nitrogen dynamics of the filament. Experimental data obtained from a mutant strain harboring a P*hetR::gfp* reporter cassette (Asai et al., 2009) present delayed heterocyst differentiation, observing the first heterocysts at 63–65 h after nitrogen deprivation instead of the typical 18–24 h (Flores and Herrero, 2010), indicating that the differentiation process is somehow altered in this strain. In their model, the authors continued the idea, first presented by Baker and Herman (1972), that cell division and heterocyst differentiation are two competing mechanisms. They proposed a cellular automaton model where cells have the capacity of aging, dividing, and differentiating into heterocysts (that are immediately functional), and dynamics are simulated with a Gillespie algorithm (Gillespie, 1976). The division and differentiation probabilities are represented by sigmoidal Hill functions of the cell age. Additionally, the differentiation rate is affected by a lateral inhibition produced by existent heterocysts. This effect decreases as the number of vegetative cells to the source heterocysts increases. The initial condition for the simulation is a filament of a random number of cells with random ages flanked by two heterocysts. The model reproduces the experimental distribution of segments between heterocysts but not the age distribution of the cells that differentiate. However, it is worth noting that the filaments in which all vegetative cells differentiate into heterocysts before the filament has grown up to 5,000 cells are discarded. The model predicts that most cells differentiate at an older age, while experimentally, the differentiation happens at a younger age. From this, it is inferred that the model does not properly capture early pattern formation. To solve this disagreement, *hetR* transcription was studied, observing that it was not immediately perturbed by cell division and remained active at the early stage, concluding that *hetR* activity should be considered independent of cell age. Following this, a model was presented, where differentiation is independent of cell age, obtaining a more realistic age distribution of the commitment time. Finally, both early (defined as 63–65 h after nitrogen deprivation) and late (more than 69 h after nitrogen deprivation) differentiation could be explained with the same kinetic parameters by altering the differentiation dependency with cellular age. Given that the commitment time to differentiation is around 7–8 h (Yoon and Golden, 2001; Muro-Pastor and Hess, 2012), it is evident that the reporter strain used introduces artifacts, and any conclusion based on its observation has to be taken with extreme caution.

All the models discussed up to this point are remarkably capable of reproducing the overall experimental interval length distribution of heterocysts; however, they fail to capture the early pattern formation in the filament. In addition, the initial conditions for almost all these studies are filaments with functional heterocysts in the extremes. Therefore, since inhibitors will only reach the cells close to a heterocyst, only the regions far from these heterocysts, if long enough filaments are considered, would properly reflect *de novo* pattern formation. Additionally, all these models only consider an inhibitory signal originating from the heterocysts without including the well-known competitive lateral inhibition between vegetative cells through PatS (Yoon and Golden, 2001; Corrales-Guerrero et al., 2013; Du et al., 2020). Given that the only selection mechanism acting over the vegetative cells during the first round of differentiation is the initial heterogeneity, the authors are forced to add arbitrary mechanisms to avoid the excessive simultaneous differentiation of contiguous cells. In the studies by Allard et al. (2007) and Brown and Rutenberg (2012a,b), the mechanism is an immediate big release of nitrogen from the heterocysts that stops the differentiation of the close neighbors of committed cells. Alternatively, in the study by Ishihara et al. (2015), the model is fitted with a strain with an apparent differentiation impairment in which the first round of differentiation appears almost three times later than the typical appearance time.

As a result of these limitations, it seems necessary to include an inhibitory signal originating from vegetative cells in order to fix the heterocyst pattern. Thus, once this initial pattern is formed, an inhibitory signal originating from the heterocysts, which could be due to the fixed nitrogen (Fogg, 1949; Water and Simon, 1982), to a paracrine inhibitor identified as HetN in Callahan and Buikema (2001) or to a combination of both, could be enough to maintain the preexisting pattern.

## 3 Genetic regulatory models

**FIGURE 2:**
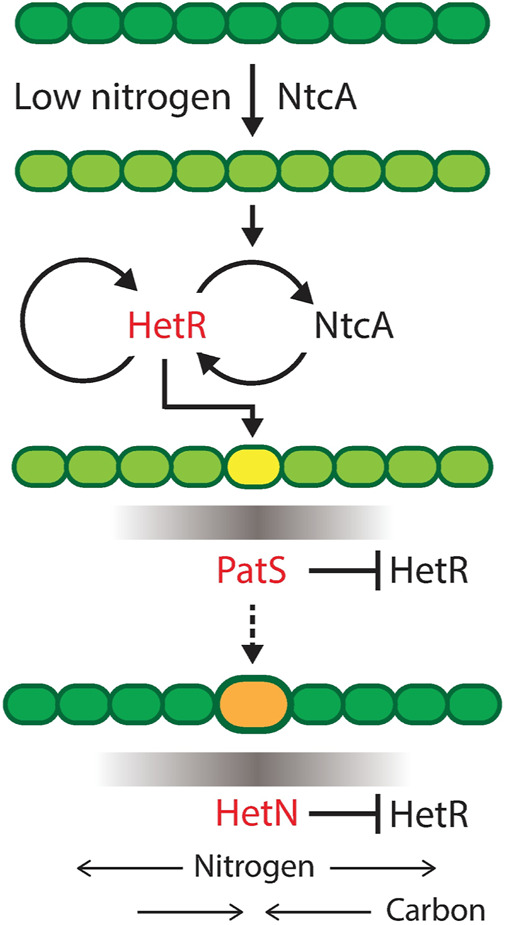
Progress of heterocyst differentiation. The scheme represents the process of differentiation of a filament of heterocyst-forming cyanobacteria with the roles of the main genes involved. Darker green means more fixed nitrogen in a cell. Low nitrogen induces NtcA expression, which, in turn, activates HetR, the master regulator of heterocyst differentiation. Yellow is a cell committing to differentiation and producing a gradient of PatS inhibiting the action of HetR in neighboring cells. Orange is a differentiated cell, a heterocyst, producing fixed nitrogen and a gradient of the inhibitor HetN and receiving carbon from vegetative cells. Reproduced from Di Patti et al. (2018), CC BY 4.0 license.

The role of the main genes involved in heterocyst differentiation is depicted in Figure 2. The differentiation mechanism is initiated by the upregulation of *ntcA* in nitrogen deficiency conditions. This increase of *ntcA* causes an increase of *hetR* that initiates the production of *patS*. This gene codifies a lateral inhibitor that avoids the differentiation of several contiguous cells into heterocysts. Once the cell has already differentiated, it starts producing both fixed nitrogen and *hetN,* which is another inhibitor of heterocyst formation. *hetR* is the master regulator of the process: in its absence, there is no heterocyst differentiation, consistent with observations in *Cylindrospermopsis*, which is the only Nostocal that lost the ability to develop heterocysts and fix nitrogen.

**FIGURE 3:**
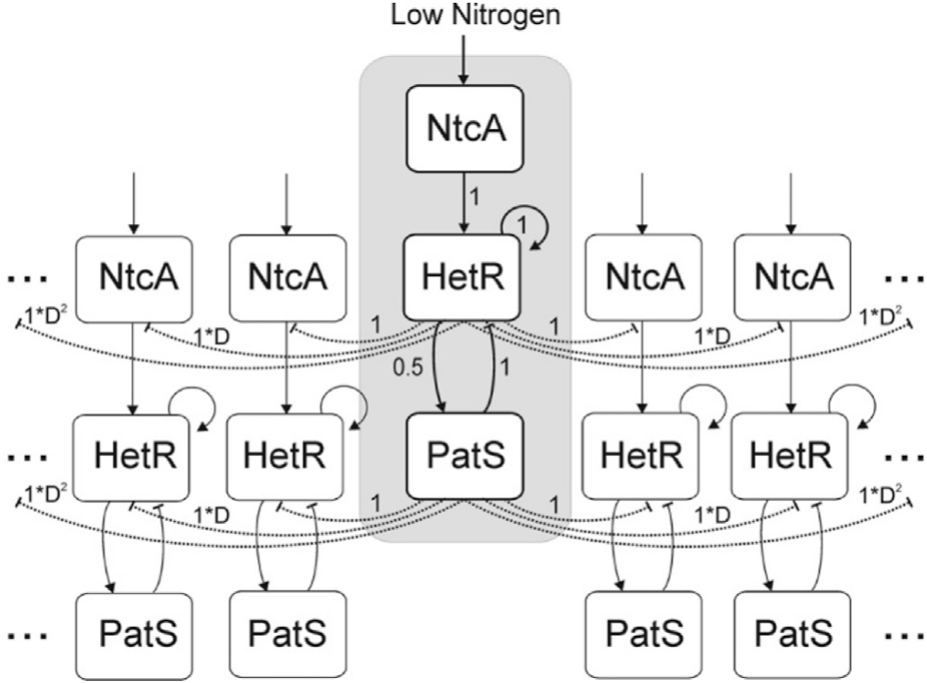
Diagram of the network considered in the study by Gerdtzen et al. (2009). Cells are organized in a cyclical manner. Direct interactions are represented by solid lines and, indirect interactions are represented by dashed lines. Arrow heads indicate activation, and vertical lines indicate inhibition. Numbers indicate the strength of the interactions considered among the elements of the network. Reproduced from Gerdtzen et al. (2009), CC BY 2.0 license.

In the study by Gerdtzen et al. (2009), a deterministic compartmental model was introduced with three genes represented by a vector, with values in the interval [0, 1], that interact between them through an interaction matrix. The genes considered are *ntcA* and *hetR*, and *patS* and *ntcA* are considered to be activated by nitrogen depletion (Vega-Palas et al., 1992) and, in turn, activate *hetR* (Muro-Pastor et al., 2002). *hetR* is considered to activate both itself and *patS* (Huang et al., 2004). Finally, *patS* inhibits *hetR* production (Yoon and Golden, 1998). All these interactions are considered to have the same relative strength, except the *hetR* activation of *patS* which is defined to have half of this strength. An explicative diagram of the model is included in Figure 3.

This model also includes a proxy for *patS* and fixed nitrogen diffusion through a multiplicative factor *D* ^*n*^ (*D* < 1). This factor reduces the inhibitory effect of *patS* over the *hetR* expression of a cell located *n* cells away from the *patS* source. The inhibitory effect of *ntcA* through fixed nitrogen is characterized as an inhibition from *hetR* expression, given that the cells with *hetR* = 1 will be considered heterocysts.

The simulation is initialized from random conditions, and then state transitions are considered to occur asynchronously, with one gene state on a given cell being updated using the interaction matrix at a time in random order for the whole array of cells. After a certain time, the system converges to a patterned filament, where $\bar{L_{H}}$ , the average interval between heterocysts (cells expressing all genes in the model at the maximum possible level, 1) depends on the value of the diffusion constant *D*. Increments of *D* up to a critical value of 0.7 produce an almost linear increase in $\bar{L_{H}}$ due to the creation of fewer heterocysts. However, from this point onward, the behavior of $\bar{L_{H}}$ stops being linear, and the system saturates to a state without any heterocyst for *D* ≥ 0.92.

The authors set the value of D that produced an $\bar{L_{H}}=10\pm 2$ cells, which is similar to the experimental value observed by Yoon and Golden (1998) to study the system. They presented the histograms for intervals between heterocysts in the case of the wild type, the *patS* deletion mutant, and the *hetR* over-expressed condition. These results show that, while the means $\bar{L_{H}}$ are compatible with the experimental data, the simulation produces histograms much more skewed towards larger intervals for both the wild type and the over-expression of *hetR*, and a strictly decreasing distribution of interval length for the *patS* mutant. The first discrepancy could be caused due to the reinterpretation of the fixed nitrogen inhibition of *ntcA* through *hetR*. This change would produce an additional inhibitory signal originating from developing cells instead of only from mature heterocysts as it should be. On the other hand, the discrepancy in the *patS* mutant could be just a problem of interpretation. Given that the expression of the variable *patS* is never shut off, one could argue that this variable is a joint representation of the two main inhibitory genes, *patS* and *hetN*. Then the deletion of this variable should result in the complete differentiation observed in the double Δ*patS*Δ*hetN* mutant (Borthakur et al., 2005), but with the additional artificial inhibition of *hetR* described earlier. This inhibition partially rescues this mutant because it fulfills the same dual role. The rescue is not full because it targets *ntcA* instead of *hetR*, which reduces its efficiency.

A continuous representation of a linearly growing one-dimensional filament was presented in Zhu et al. (2010). The system of equations that defines its dynamics is as follows:

$$\frac{dr}{dt}=\alpha_{r}+\beta_{r}F\left(r,s\right)+G\left(r,s,n\right)-\kappa_{r}r$$ (2)

$$\frac{ds}{dt}=\alpha_{s}+\beta_{s}F\left(r,s\right)+D_{s}\frac{\partial^{2}s}{\partial x^{2}}-\kappa_{s}s$$ (3)

$$\frac{dn}{dt}=\beta_{n}F\left(r,s\right)+D_{n}\frac{\partial^{2}n}{\partial x^{2}}-\kappa_{n}n,$$ (4)

where *r* is the concentration of HetR, *s* of PatS, and *n* of HetN. The spatial domain, that is, the filament length *L*, grows at a constant rate *ρ*:

$$\frac{dL}{dt}=\rho L.$$ (5)

This model considers linear degradation rates (*κ*_*r*_, *κ*_*s*_, *κ*_*n*_) for all the proteins and diffusion of the two inhibitors with rates *D*_*s*_ and *D*_*n*_. Regarding protein production, the authors considered basal production for both HetR (*α*_*r*_) and PatS (*α*_*s*_) and regulated production for all genes through the function

$$F\left(r,s\right)\equiv \frac{r^{2}}{\left(K_{s}+s\right)\left(K_{r}^{2}+r^{2}\right)},$$ (6)

and an additional production term for *hetR*.

$$G\left(r,s,n\right)\equiv {\left(r_{e}-r\right)}^{2}\left(n_{c}-n-\eta s\right).$$ (7)

Both Eqs 6 and 7 include the HetR homodimer formation described in Huang et al. (2004) through a quadratic *hetR* variable. Eq. 6 models activation of HetR, PatS, and HetN by HetR dimers and inhibition by PatS. Eq. (7) is a phenomenological term affecting HetR: its strength depends on the difference between HetR concentration and an ad hoc level *r*_*e*_, and its sign is set by the parameter *n*_*c*_: when the combination of HetN and Pats concentrations given by *n* + *ηs* is larger than *n*_*c*_, function *G*(*r*, *s*, *n*) has the effect of a degradation; otherwise, it promotes the production of HetR. Through this term, low levels of inhibitors have the effect of an extra activation that disappears only when the concentration of HetR is *r*_*e*_. With this model, the goal is to study pattern maintenance; due to this, the initial condition simulates the presence of heterocysts in the borders of the system. This condition is translated into a uniform initial distribution of both HetR and PatS, set to their equilibrium concentration based only on the constitutive production and degradation terms; in the heterocysts, the concentration of HetR is set to the equilibrium value *r*_*e*_. HetN is initially set to a diffusion-mediated “bowl-shaped” distribution, with the maxima at the heterocysts. In a way akin to Turing patterning (Turing, 1952), the apparition of only one heterocyst in the middle is heavily conditioned by the difference in the two inhibitors’ diffusive rates. Particularly, the diffusion of HetN should be lower than the filament growth rate so that there can be HetN depletion in the middle of the filament to induce HetR production. The diffusive rate of PatS must be higher than the one of HetN to reduce the length of the induced region. With these conditions, the model properly reflects the rise of HetR in the middle of the filament that is hypothesized to originate from the new heterocyst (Black et al., 1993) and the reported inhibitory gradients produced by it (Risser and Callahan, 2009).

Low robustness of the pattern to modification of the diffusion parameters is characteristic of Turing-like continuous models. It stems from the requirement that the pattern is an equilibrium state of the overall regulatory system, and consequently, the interplay of the two inhibitors must be tuned in such a way that the range of the inhibitors is different enough to create steady spatial differences in gene expression that originate the pattern. However, discrete systems such as *Anabaena* filaments can fixate an unstable pattern through the irreversible commitment of a cell that presents a sustained high expression of a given gene, even if that expression is transient and would be reversed without the differentiation. For this reason, in *Anabaena* dynamical stability of the pattern is much less relevant than its establishment.

Brown and Rutenberg (2014) presented the last model of the series discussed in the previous section on diffusion models. In this study, we incorporate a mechanism of genetic inhibition into the nitrogen diffusion model presented by Brown and Rutenberg (2012a). This lateral inhibition through *patS* and *hetN* substitutes the immediate release of nitrogen and allows a more biologically realistic maturation of the heterocysts. Both genes are modeled as Boolean variables that directly prevent the commitment to differentiation of a fixed range of contiguous cells. To replicate the experimental observations, this range is set to five cells. The expression of both *patS* and *hetN* is, in turn, modeled as deterministic switches.

On the one hand, *patS* inhibition starts right after commitment until the complete maturation of the heterocyst (10 h after commitment), and a time *τ*_*S*_ (set to 1 h) after this point, the heterocyst starts producing fixed nitrogen. On the other hand, *hetN* inhibition starts a certain time *τ*_*N*_ (also set to 1 h) after commitment and is never shut off.

The initial condition considered is a lonely cell, which grows for over 7 days in nitrogen-rich conditions in order to get a heterogeneous filament, which will be put under nitrogen-deprived conditions. The model properly reproduces the vegetative interval histograms tendencies for all the mutants but with less noise and without the experimental preference for even-numbered vegetative intervals. Additionally, the authors observe that younger cells are more likely to differentiate, especially on the first round of differentiation (24 h), suggesting an indirect effect of the cell cycle on heterocyst commitment. This work shows that a deterministic model whose only random variable is the growth rate can reproduce some pattern features observed experimentally.

While the Boolean switch-like genetic model is able to reproduce the experimental mutant behaviors, it does it artificially with an immediate complete inhibition over a fixed range. This is arguably hard to justify experimentally. Despite incorporating both *patS* and *hetN* in the model, their roles are completely equivalent to the immediate release of nitrogen presented in their previous works (Allard et al., 2007; Brown and Rutenberg, 2012a,b). Instead of having distinct roles in the pattern formation, one in the pattern formation and the other in its maintenance, as hypothesized by Callahan and Buikema (2001), they are modeled with the same function (which is to avoid the formation of multiple heterocysts). Additionally, the design of the switch-like dynamics forces the mutant phenotype by providing a window of a duration *τ*_*S*_ (after the cell commitment) and *τ*_*N*_ (after *patS* deactivation) in which there is no inhibition of differentiation in the system for the mutants Δ*patS* and the Δ*hetN*.

**FIGURE 4:**
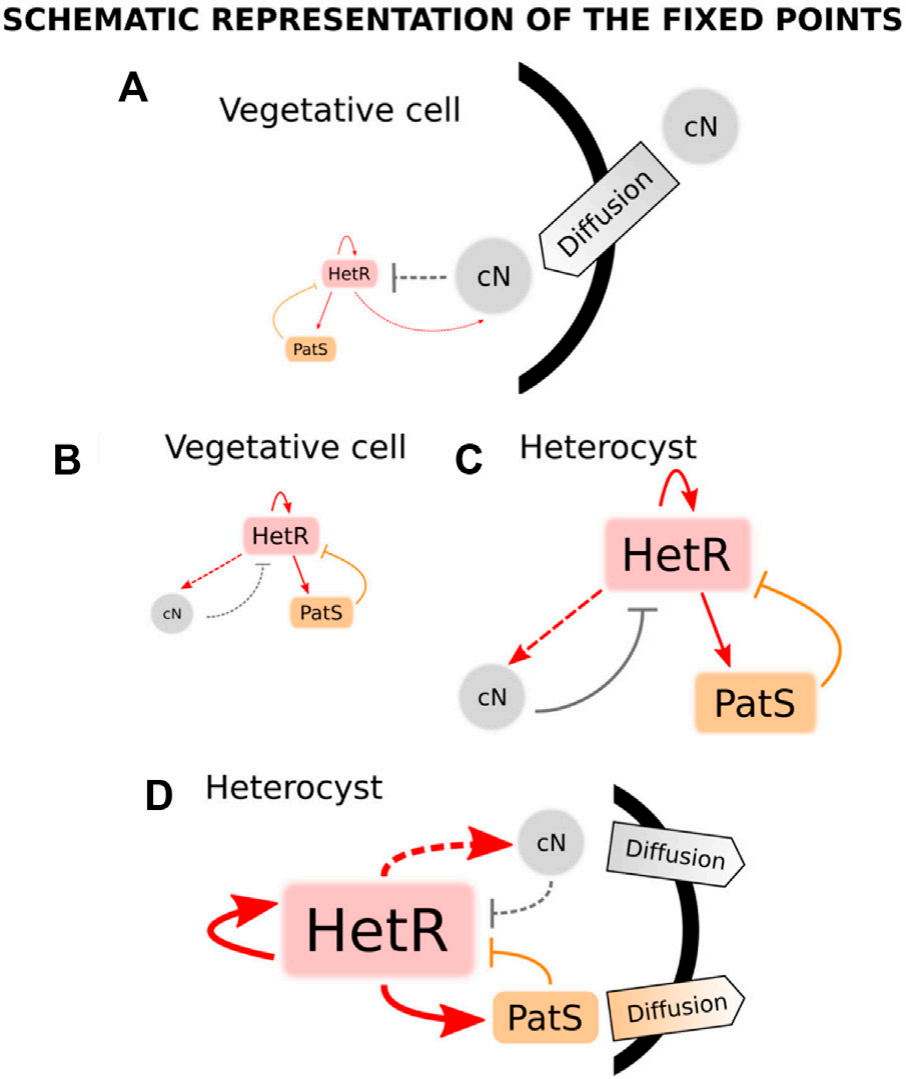
States of a cyanobacterium when subjected to different conditions of nitrogen and diffusion in the model in the study by Torres-Sánchez et al. (2015). When combined nitrogen (cN) is provided to the cell, there is only one stable fixed point **(A)**, which corresponds to a state in which the production of both HetR and PatS is minimum (vegetative state). When subjected to nitrogen deprivation, there are two stable fixed points **(B and C)**. The first point **(B)** is a vegetative state in which there exists an equilibrium between a small production of HetR, PatS, and cN. The same kind of equilibrium is present in the second fixed point **(C)** but in this case, the production of all transcription factors and cN is high (heterocyst steady state). When the cell is exposed to nitrogen stress, its trajectory evolves from **(A)** to the steady state **(B)** and, thus, it remains vegetative. Assuming some diffusion of cN and PatS from the cell, the only stable state **(D)** corresponds to a heterocyst state with high levels of production of HetR, cN, and PatS, being the latter transported to the surroundings of the cell. Adapted from Torres-Sánchez et al. (2015), CC BY 4.0 license.

A year after this work, a model was presented that used the systems biology framework to study both the stable states of a unicellular system and the pattern formation in a filament (Torres-Sánchez et al., 2015). This model incorporates the nitrogen sensing module of the genetic network with the inclusion of the *nctA* dependence of the GS/GOAT cycle (Muro-Pastor et al., 2001) and *patS-*mediated inhibition. Particularly, *ntcA* production is increased by both HetR and NtcA and inhibited by fixed nitrogen. *hetR* transcription has the same regulation as *ntcA* plus the inhibition from *patS*. In addition, both *patS* and fixed nitrogen are positively regulated by HetR. This model considers the dynamics of fixed nitrogen, NtcA, HetR, and PatS concentrations. The authors use both the biological information of the genetic network and statistical mechanics analysis to obtain the regulatory equations of the system. After obtaining a set of parameters that reproduces heterocyst differentiation, the authors assume that there are two temporal scales: one fast, formed by HetR and ntcA, which relaxes to its steady-state much earlier than the slow one, which is due to the dynamics of PatS and fixed nitrogen. Assuming this, one can use an adiabatic argument and consider that the fast variables are in equilibrium when considering the dynamics of the slow ones. Bifurcation analysis for this reduced system is presented: for nitrogen-rich conditions, the system only presents one stable solution, which corresponds to the vegetative state (Figure 4A: low expression of both *hetR* and *patS*). On the other hand, for nitrogen-deprived conditions, the system presents bistability, with a vegetative stage (Figure 4B: with equivalent expressions of *hetR* and *patS*, but higher expression than in nitrogen-rich conditions) and a heterocyst stage (Figure 4C: with high expressions of *hetR*, *patS*, and fixed nitrogen). Additionally, the system presents hysteresis, so after nitrogen deprivation, the system will stay in the vegetative state unless a perturbation pushes it into the heterocyst state. Such a perturbation occurs when considering the diffusion of fixed nitrogen and PatS, which is enough to destabilize the vegetative state and push the dynamics to the heterocyst state (Figure 4D). The study is later expanded to a discrete filament of cells to show that by adding uniform white noise and diffusion of both PatS and fixed nitrogen, the model is capable of forming a patterned differentiation. It is also stated that the appearance of differentiation is considered a pure stochastic event and also that the biological parameters of the model can be tuned to observe the same pattern with different amplitudes of the white noise.

The high dependency on the noise to differentiate seems to contradict previous works that considered deterministic models of nitrogen-mediated inhibition (Brown and Rutenberg, 2012a, 2014) or even the same regulatory network (Gerdtzen et al., 2009). Those models produced a comparable agreement with the experimental data with much lower relevance of the noise; the stochasticity is only present in the initial conditions of the systems and not in their dynamics. In our opinion, this discrepancy can be attributed to the overlapping of the roles of both PatS and fixed nitrogen which saturates the system with inhibitors that stop the increase in HetR production. Particularly, given that the filament model is able to produce fixed nitrogen at a low level once HetR concentration rises in vegetative cells, there is no need for a transition to the high nitrogen production cellular state (heterocyst). Instead, the biological system is not capable of fixing nitrogen until the transition has already occurred, given that nitrogen fixation cannot coexist with photosynthesis. Therefore, the activation of *hetR* transcription through *ntcA* cannot be shut off until nitrogen is provided to the system. Due to this, the system is forced to maintain the individually unstable state of high expression of both *hetR* and *patS* until a heterocyst is formed in the filament. Additionally, recent experimental works (Higa et al., 2012; Corrales-Guerrero et al., 2013; Rivers et al., 2014, 2018) seem to indicate that both *patS* and *hetN* require a post-translational modification to produce the inhibitory peptide. It has been suggested (Corrales-Guerrero et al., 2014b; Rivers et al., 2018) that this transformation occurs at the cell membrane during cell-to-cell trafficking. This would avoid self-inhibition from the *patS* and *hetN* produced in a given cell and, therefore, would make impossible a unicellular stability study.

A similar systems biology approach was considered in the study by Muñoz-García and Ares (2016), where an alternative three-gene minimal model was presented. This model also included both *hetR* as the main non-diffusive regulator of the system and *patS* as an inhibitor of HetR-mediated activation. Instead of the nitrogen sensing module, the model included *hetN* as an inhibitor produced in the heterocysts. Under such a condition, *hetR* activates both *patS* and its own expression, while *patS* and *hetN* (which are produced at a basal level in the heterocysts) inhibit this activation. Fixed nitrogen is included as a direct inhibitor of HetR regulation. As a substitute for the *ntcA* role as the trigger of *hetR* expression, the model includes a low basal expression of *hetR*. Using mass-action kinetics, the authors obtained a deterministic set of differential equations from the mechanistic information of these interactions. The model considers that, while HetR needs to form a homodimer to promote expression, this activation can also be inhibited with the attachment of just one inhibitor. The stochastic nature of gene expression was considered by adding noise to the equations using Langevin dynamics (Gillespie, 2000). This genetic model was introduced in a agent based simulation of a filament with inhibitor diffusion where each cell has its own noisy dynamical variables, growth rate, and thresholds for both differentiation and cell division. The model was able to reproduce the experimental distribution of vegetative intervals between heterocysts up to the third moment of the distribution for both the wild type and the Δ*patS* mutant, and gave a reasonable prediction for the Δ*hetN* mutant for which it made no comparison with experimental data.

The phenotypical reproduction by the model of the deletion mutants reinforces the role of the two inhibitory genes proposed in the study by Callahan and Buikema (2001). This model also provides additional insight into the interplay between cell division and heterocyst differentiation. Due to the similar timescale between these two processes, the noise on the cell division defines the overall behavior of the filament. If there is low noise and cells divide in a quasi-synchronous way, the filament pattern has an oscillatory behavior with an enlargement and posterior shortening of the mean distance between heterocysts. In this low noise regime, the model also recovers the larger appearance of even-numbered vegetative cell intervals characteristic of heterocyst patterns (Meeks and Elhai, 2002). Instead, for a noisier cell division, the percentage of even intervals always remains close to 50%, and the oscillatory behavior of the mean vegetative cells interval disappears.

**FIGURE 5:**
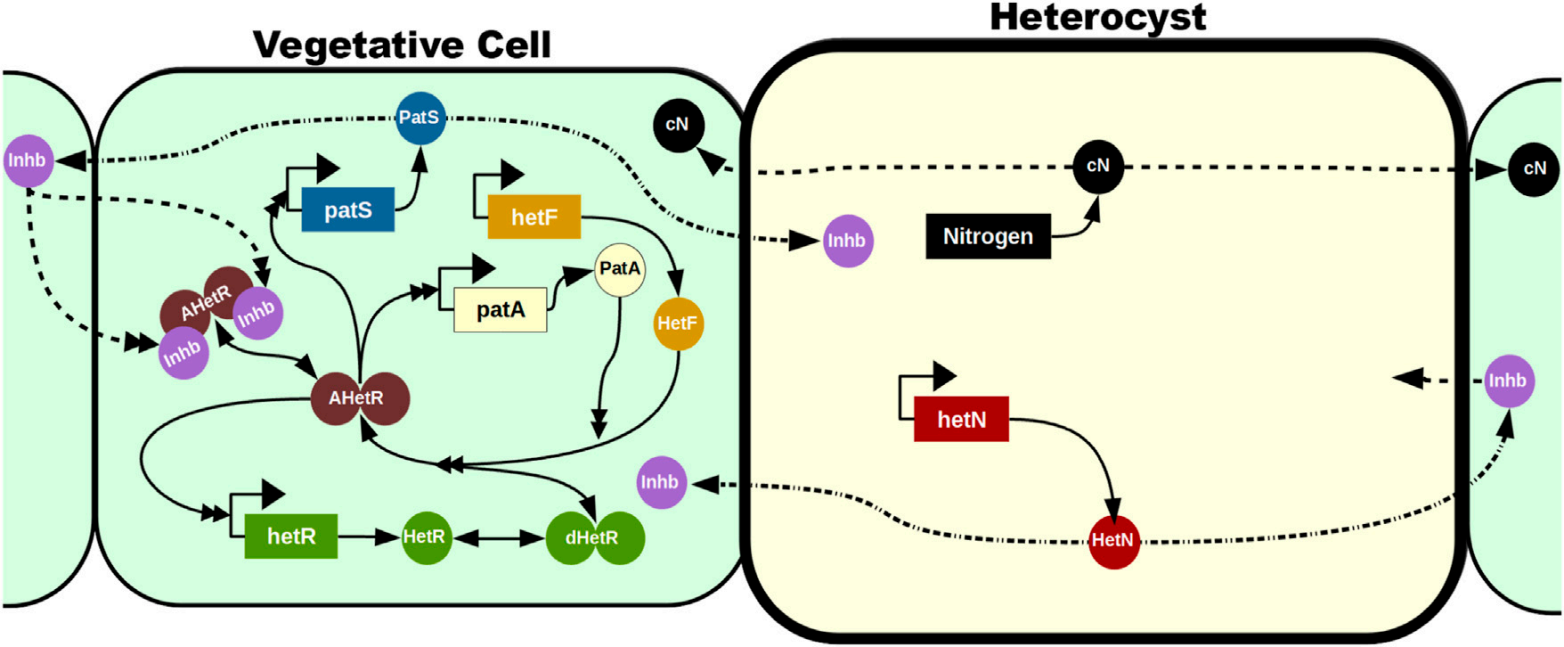
Mechanistic model in the study by Casanova-Ferrer et al. (2022). The vegetative cells are represented with a soft green background, while the heterocyst has a soft yellow background and a thicker cell wall. The reactions in the vegetative cell represent all the potential reactions in the model irrespective of the cell’s position: in most cells, only a subset of these reactions will be taking place at a given time. This subset is defined by the concentration of the inhibitor *Inhb*. Genes are represented in rectangles and proteins with circles. Dimers are represented with two attached circles and can be inactivated (green), activated (brown), and activated and inhibited (brown with two purple inhibitors attached). Solid lines represent production (with only one simple arrowhead), transformations (with a simple arrowhead on both ends), and interactions (with a double arrowhead). Dashed lines represent intercellular traffic, and dashed–dotted lines represent a transformation when exported to a neighboring cell. The figure does not represent the high expression of *hetR* in heterocysts (as presented by Corrales-Guerrero et al., 2015; Bornikoel et al., 2017; Arbel-Goren et al., 2018): since these cells have already differentiated and HetR localization is restricted to the cells where it is produced, the concentration of HetR in heterocysts is irrelevant for the model’s purposes.

A recent follow-up on this model (Casanova-Ferrer et al., 2022) includes the requirement of maturation of HetR in order to act as a transcription factor: HetF is necessary for this maturation (Risser and Callahan, 2008), and PatA enhances it (Figure 5). The product of PatS and HetN modification in the cell membrane is, for simplicity, treated as the same inhibitor (*Inhb* in Figure 5) that can be transported to neighboring cells irrespective of their nature, although only inhibition of mature HetR action in vegetative cells is explicitly modeled. This work focuses especially on the phenotype of the deletion mutant of *patA*. This mutant does not present patterning, and the appearance of heterocysts seems to be purely stochastic, with a huge preference for presenting heterocysts in the filament ends. The model is capable of reproducing this phenotype and predicts a homogenization of the HetR concentration in the filament when PatA is absent due to a reduction in the activation rate of HetR. This homogenization prevents the formation of a pattern and, therefore, internal heterocysts are formed exclusively due to random fluctuations of production rates, inhibitor diffusion, or decision mechanisms. However, if one considers some kind of leak of inhibitor through the filament ends together with the fact that the terminal cells only receive inhibitor from one neighboring cell, terminal cells present a much higher probability to differentiate than internal cells.

This analysis of the *patA* mutant is especially relevant because it is a clear example that one can disrupt the formation of the pattern by affecting the intensity of the feedback loops controlled by HetR. As *patA* is hypothesized to have a supporting role to *hetF*, one could expect that the overall behavior of the network should not be that affected. However, given that without *patA,* the fraction of HetR that gets activated is reduced with respect to the wild type, this mutant seems to lose the compounding effect that allowed the formation of the pattern. This mutant is much less susceptible to sudden spikes of HetR production, and, therefore, most of the stochastic fluctuations get buffered without affecting the overall homogeneity of the filament.

Following the Turing-like characterization, Huang et al. (2004) and Di Patti et al. (2018) present the same three-gene system (*hetR* as an activator and both *patS* and *hetN* as diffusible inhibitors), but, in this case, the inhibitory effect is produced through degradation of HetR dimers mediated by PatS and HetN. The model assumes a basal production in all cells and a linear degradation for the three genes, and an increase in the production of both HetR and PatS activated by HetR. As the model does not enforce any distinction between vegetative and heterocyst cells, all cells actively produce both inhibitors simultaneously. Thus, it does not reflect the temporal differences in the onset of production of PatS and HetN. A bit surprisingly, the same work presents very nice experimental evidence of this difference through GFP reporters of transcription.

With this set of interactions, the authors obtain a set of differential equations through the van Kampen expansion. Initially, the authors study the linear stability around the homogeneous fixed point of the mean field approximation. Through this analysis, the authors located a set of parameters that allow the formation of instabilities that could originate a pattern in the mean field conditions. As already mentioned during the analysis of Huang et al. (2004), the most relevant parameters are the diffusion constants for both inhibitors. The smaller the ratio $\frac{D_{S}}{D_{N}}$ is, the narrower is the instability region. Subsequently, the authors introduce the same interactions in a Gillespie algorithm with the same set of parameters to check how stochasticity affects pattern formation. The authors show that the presence of noise promotes the spontaneous selection of a leading wavelength in the emerging pattern. Due to this the parameter region where the system presents instabilities and, therefore, pattern seeding is considerably larger in the noisy system. The addition of filament growth (cellular division) to the model increases the amount of available unstable modes of the system. Despite this similarity, the patterning is much more stable in the system with deterministic growth than in the noisy one. Without noise, a new high HetR expression region (heterocyst) appears in the midsection of the existing pattern when the filament elongates enough. On the other hand, the addition of growth to the noisy system destabilizes the patterning and allows for the transition between the different unstable modes that arise with the filament growth. This implies that while the pattern formation is enhanced by the addition of noise to the system, its maintenance in a growing domain requires an irreversible fixation of the heterocyst state. Additionally, this model shows that it is possible to form the pattern through the regulation of protein degradation instead of the previously studied regulator inhibition. Nevertheless, it should be tested if this alternative inhibition through HetR degradation reproduces the experimental data for a model with a more realistic temporal separation between the two inhibitors.

After this systematic analysis of the existent genetic models, one can extract some common key ideas. First, the realization that several different configurations of a minimal three-gene network with an activator, *hetR*, and a couple of inhibitors, *patS* and typically *hetN*, but it could also be the fixed nitrogen through *ntcA* regulation, as in Gerdtzen et al. (2009), are capable of reproducing the wild type behavior. Due to this, it seems indispensable to consider other conditions, especially the deletion ones, in order to properly evaluate the regulatory mechanisms proposed. There must be a certain temporal separation between the inhibitory effects in order to originate a pattern. This difference could be either produced due to the relationship between the diffusion coefficients (Zhu et al., 2010; Di Patti et al., 2018), directly imposed (Brown and Rutenberg, 2014) and also (Muñoz-García and Ares, 2016; Casanova-Ferrer et al., 2022) (where *hetN* is exclusively produced in heterocysts), or in the case of fixed nitrogen (Gerdtzen et al., 2009; Torres-Sánchez et al., 2015) acting indirectly through *ntcA* and, therefore, presenting a certain delay.

## 4 Cyanobacteria population models

An alternative point of view to the study of spatial pattern formation is to consider the cyanobacteria culture as a population problem where the percentage of each cell type is defined by external conditions.

This approach is used in the study by Hense and Beckmann (2006), presenting a deterministic model of the life cycle of cyanobacteria dependent on energy, mainly in the form of light and nitrogen availability. In this formulation, the heterocyst would be the stage with high energy (abundant light) and low nitrogen availability. The model is capable of reproducing the seasonal changes in the cyanobacteria population composition and infers a correlation between summer blooms and cycle velocity, where previous summer conditions strongly affect the possibility of explosive growth. The scope of this work is mostly ecological and does not provide an extensive insight into the mechanisms controlling the vegetative-heterocyst transition.

Alternatively, Pinzon and Ju (2006) take the same culture level population approach but with a more biomolecular focus on the cellular processes that modulate the transition from a vegetative cell to a heterocyst. The deterministic model proposed includes photosynthetic growth of vegetative cells, heterocyst differentiation, self-shading effect on light penetration, and nitrogen fixation. The authors hypothesize that heterocyst differentiation is driven by the difference between the required fixed nitrogen to support maximal growth and the available nitrogen. The model describes experimental profiles well and gives reasonable predictions even for the transition from growth over external nitrogen sources to self-sustained growth.

This population point of view was taken again later by Grover et al. (2019). In this work, the authors present a deterministic model where the transition between vegetative and heterocyst cells is controlled by the relationship between the processed and free concentration of both nitrogen and phosphorus in the cells. The model predicts a relationship between the heterocyst to vegetative ratio with the nitrogen to phosphorus ratio of the environment. The authors use this to discuss an evolutionary reason for the regulation of heterocyst differentiation. Given that phosphorus-limited habitats are much more common than nitrogen-limited ones, the costly investments in nitrogen fixation are tightly regulated.

As one can see, this kind of mean-field point of view is more useful for an ecological and evolutionary perspective but does not provide much insight into the regulation of heterocyst differentiation. The patterned differentiation of heterocysts seems relevant to the mechanism controlling the differentiation decision; therefore, the population point of view is less optimal because the pattern information is lost.

## 5 Conclusion

**FIGURE 6:**
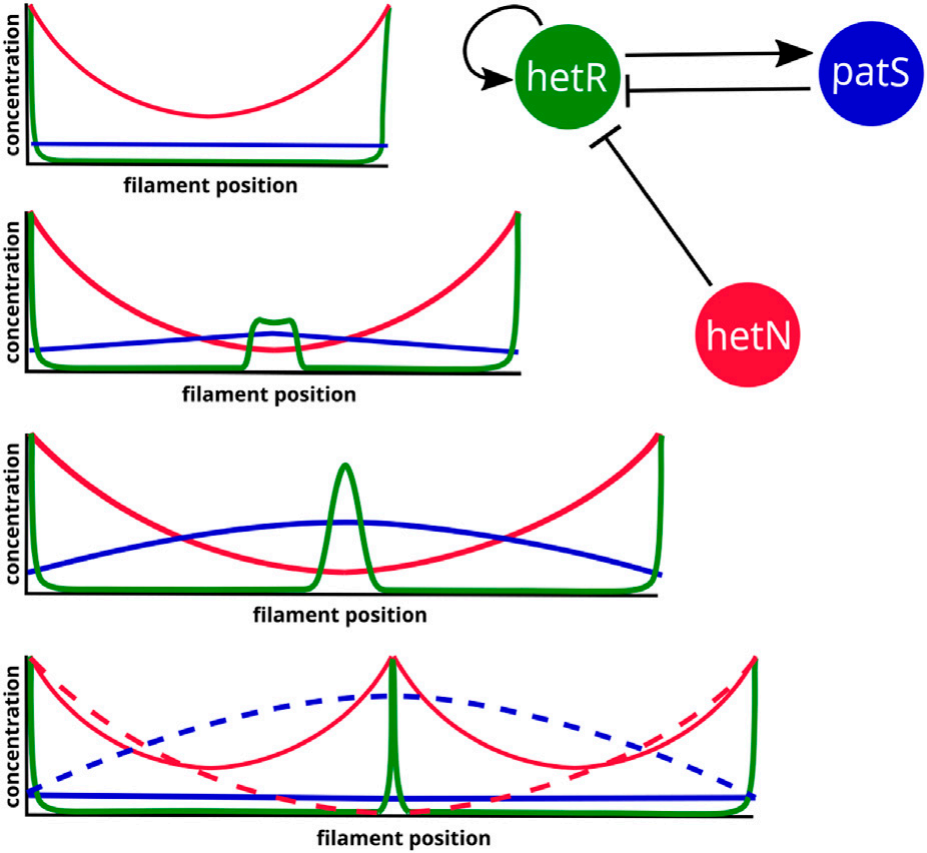
Three-gene simplified regulatory system and expected protein concentration dynamics for the emergence of a new intercalary heterocyst in a fully deterministic system. The heterocysts are represented as peaks of HetR that produce HetN. The dashed profiles represent the state right before the transition to a heterocyst.

In the section on inhibitor diffusion models, we have discussed examples of models where just a diffusible inhibitory signal produced in the heterocysts is enough to maintain an existing pattern in a filament. If one considers the genetic regulatory system (Figure 2), it is easy to see that the role of this inhibitory signal originating from the heterocysts would be taken by HetN and fixed nitrogen. HetN acts directly over HetR and fixed nitrogen indirectly through *ntcA*. With this minimal structure a new heterocyst would arise in the space between heterocysts roughly when the interval doubles its length. Then, as observed when discussing genetic regulatory models, if one also considers *patS*, which is a lateral inhibitor expressed in vegetative cells, the system is capable for creating *de novo* pattern formation. This regulatory system (Figure 6) would be coupled with a switch-like genetic mechanism that initiates differentiation when the HetR concentration is higher than a certain threshold. The three-gene system of an activator and two inhibitors could seem like a Turing pattern, but it presents a key difference, one of the inhibitors *hetN* has its production restricted to the heterocysts. Moreover, this differentiation to heterocysts entails a morphological change and, therefore, is irreversible. This ensures the stability of the pattern that would not be possible in a Turing system.

**FIGURE 7:**
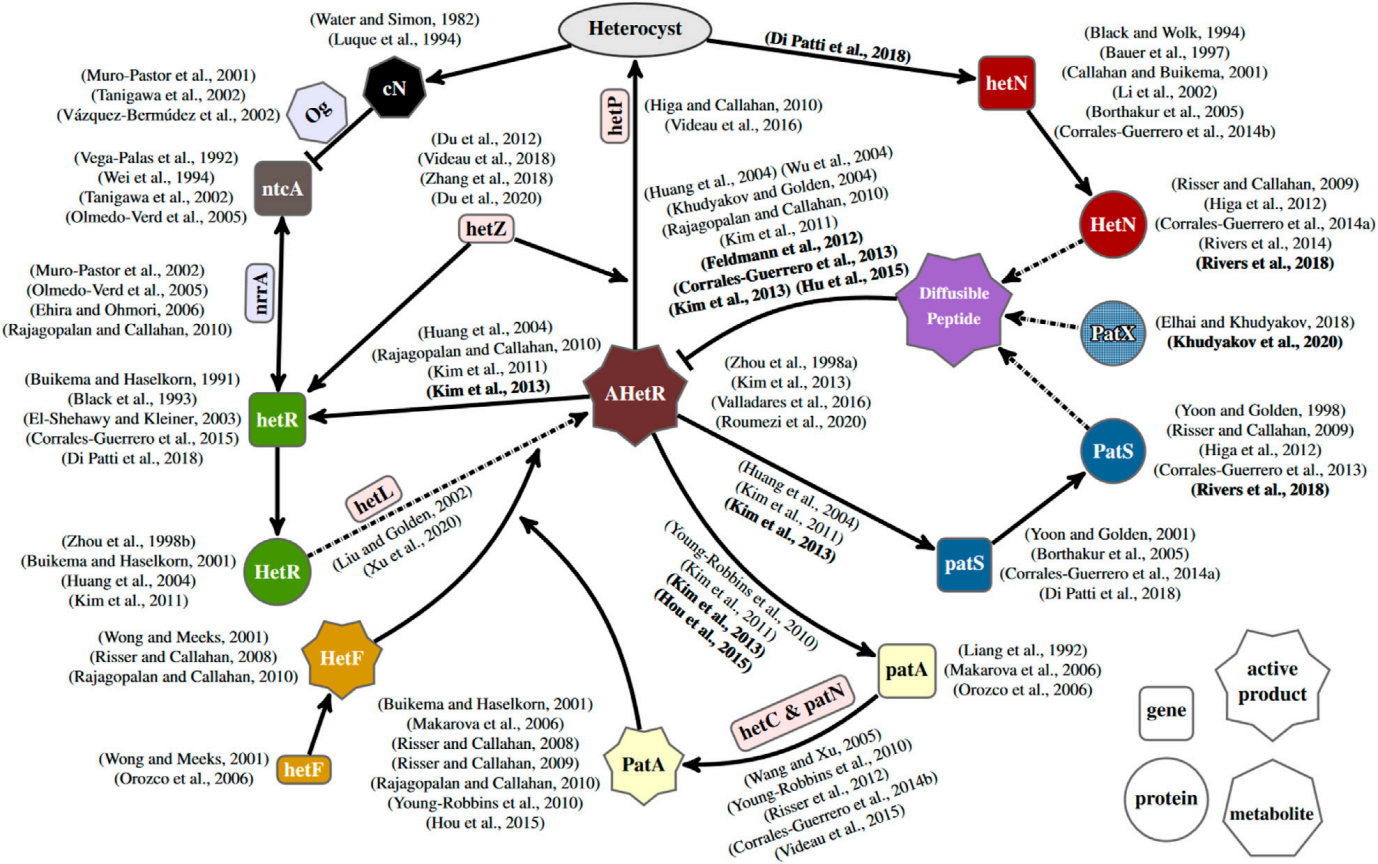
Gene regulatory network of heterocyst differentiation. The main elements and their interactions are depicted schematically, together with other relevant elements with either more dubious (in light red background) or oversimplified roles (in light blue background) in the scheme. AHetR stands for the active form of HetR. The ellipse represents the differentiation into a heterocyst. Arrows with solid lines represent interactions between elements. Arrows with dashed–dotted lines represent post-transcriptional changes. Regular and bold formatted references indicate phenotypically inferred and observed molecular interactions, respectively. Discussing all the references contained in this figure, which describe experimental results, is out of the scope of this work. For this reason, a number of them are not cited in the text.

Different strategies to model these three genes can simulate a heterocyst pattern, so more biological information is necessary to properly define the differentiation mechanism. A powerful tool is constraining models by comparison with different genetic backgrounds. Figure 7 shows a simplified regulatory network of heterocyst differentiation obtained from surveying experimental literature. From this, it is evident that the mechanism controlling heterocyst differentiation is quite more complex than any model discussed in this work. Therefore, incorporating more genes into the models would, on the one hand, deepen the understanding of the regulatory network and, on the other hand, open the possibility to compare with a wider range of genetic backgrounds. Therefore, the way forward is to incorporate into models genes that still have dubious roles, represented in light red rectangles in Figure 7. The research on those genes is still quite brief, and there is not enough information to justify their inclusion in models. There is evidence that both *hetC* and *patN* are connected to *patA* regulation, but there is not enough information to assign a proper role to them. On the other hand, the function of *hetL* seems quite clear: it appears to be involved with HetR activation, but there is no clear link to *hetF* and other genes in the system other than *hetR*. Also, with an apparently clear function but without a clear relation with the other genes are both *hetP* and *hetZ*, which are heavily linked to the heterocyst commitment but without a clear explanation on how they affect the commitment. This patched information is natural given that usually, the first experimental evidence is the effects of the knock-out mutants over the known network. Posterior studies that provide experimental information regarding protein translation, such as studies by Corrales-Guerrero et al. (2015) and Di Patti et al. (2018), could be really useful to properly include the gene in a model of the regulatory network. Modeling of putative mechanisms for these genes could be a useful source of predictions, helping to focus on what experiments should look for. An example is the prediction of inhibitor leakage at the filament extremes as necessary to explain the *patA* phenotype (Casanova-Ferrer et al., 2022). This expansion of the genetic scope of the models would bridge the gap that now exists between this kind of reduced model focusing on heterocyst differentiation and the more general genome-scale frameworks, such as the study by Malatinszky et al. (2017), which model the full metabolism of the *Anabaena* cell and how it changes after the differentiation into a heterocyst.
